# Diagnosis and management of keratoconus by eye care practitioners in Kenya

**DOI:** 10.1186/s12886-023-02792-w

**Published:** 2023-01-27

**Authors:** Zahra Aly Rashid, Vanessa R Moodley, Khathutshelo Percy Mashige

**Affiliations:** School of Health Sciences, Discipline of Optometry, University of KwaZulu-Natal, Private Bag X54001, Durban, 4000 South Africa

**Keywords:** Keratoconus, Vernal keratoconjunctivitis, Optometrist, Ophthalmic clinical officers, eye-care guidelines, Africa, Kenya

## Abstract

**Background:**

To explore current eye care practice in keratoconus diagnosis and management in Kenya.

**Methods:**

An online questionnaire was distributed to ophthalmic clinical officers (OCO) and optometrists.

**Results:**

A total of 203 responses were received from 52 OCOs and 151 optometrists with a response rate of 24.4% and 53.5% respectively. The majority reported having access to retinoscopes (88.5%; *p* = *0.48*) and slit lamps (76.7; *p* = *0.14*). Few practitioners had access to a corneal topographer (13.5%; *p* = *0.08*) and rigid contact lens (CL) fitting sets (OCOs 5.8%, optometrists 33.8%; *p* < *0.01*). One-third did not feel that retinoscopy (38.7%; *p* = *0.21*), slit lamp findings (30.3%; *p* = *0.10*) and corneal topography (36.6%; *p* = *0.39*) are important investigations in keratoconus diagnosis. Corneal topography was not recommended in two-thirds of patients (59.0%; *p* = *0.33*) with vernal keratoconjunctivitis (VKC). The majority counselled against eye rubbing in mild (73.6%; *p* = *0.90*) VKC, 52.9% in moderate (*p* = *0.40*) and 43.6% in severe (*p* = *0.24*) cases. The majority prescribed spectacles in mild (90.2%; *p* = *0.95*), 29% (*p* = *0.97*) in moderate and 1.9% (*p* = *0.05*) in severe cases. When the binocular best corrected visual acuity (BCVA) with spectacles was ≤ 6/18, 76.9% of OCOs and 58.9% of optometrists referred for CLs (*p* = *0.02*). When binocular BCVA with CLs dropped to ≤ 6/18, 83.7% (*p* = *0.18*) referred to the ophthalmologist for surgical intervention. Few OCOs fitted rigid CLs (15.4% OCOs, 51.0% optometrists; *p* = *0.01*), majority referred to optometrists (82.7% OCOs, 43.7% optometrists; *p* < *0.01*). Progression was monitored in 70.1% (*p* = *0.11*) of mild, 50.9% (*p* = *0.54*) moderate and 25.3% (*p* = *0.31*) advanced cases. Few OCOs (15.4%) performed corneal cross-linking (CXL). A few respondents (5.4%; *p* = *0.13*) did not know when to refer keratoconus patients for CXL. Co-management with ophthalmologists was reported by 58.0% (*p* = *0.06*) of respondents.

**Conclusion:**

The results of this study highlight the need to map services for keratoconus patients, review current curricula and continuous education priorities for mid-level ophthalmic workers, develop guidelines for the diagnosis and management of keratoconus and improve interdisciplinary collaboration.

**Supplementary Information:**

The online version contains supplementary material available at 10.1186/s12886-023-02792-w.

## Background

Keratoconus is a progressive, bilateral and asymmetrical disorder, characterized by corneal steepening, protrusion, corneal thinning and irregular astigmatism [1]. It appears around puberty or in the second decade of life and generally does not progress after the age of forty [2]. It is a multifactorial disease with genetic, biochemical, biomechanical and environmental factors [3]. Down syndrome, Ehlers-Danlos syndrome, Leber congenital amaurosis, connective tissue disorders (Marfan syndrome), eye rubbing, family history, ocular allergy and atopy have been cited as the most important risk factors for keratoconus [3]. Globally, the prevalence rates of keratoconus are estimated between 0.2 and 4 790 per 100 000 persons [1]. This considerable variation is likely related to several factors, including research methodology, populations studied, and access to eye health care and diagnostic criteria. There remains a shortage of epidemiological data on keratoconus in Africa and the prevalence of the disease in Kenya is currently unknown.

The symptoms and signs of keratoconus vary depending on severity. In the early stages, it can mimic simple refractive errors with good visual acuity. As it progresses there is a reduction in best corrected visual acuity (BCVA), distorted vision and ‘ghosting’ [1]. Keratoconus is diagnosed by a combination of tools namely retinoscopy, slit lamp signs, corneal topography, pachymetry and corneal tomography. Corneal protrusion, scissors retinoscopic reflex, corneal thinning, Fleischer’s ring and prominent nerve fibers have been identified as the most prevalent findings in keratoconic corneas [4].

Disease management will depend on severity and progression; aimed at halting progression and vision rehabilitation. Nonsurgical management is with spectacles in mild cases, specialized soft and rigid contact lenses (CL) in moderate cases and hybrid/scleral CLs in severe cases. Surgical options include intra-stromal corneal ring segments (ICRS), corneal cross-linking (CXL) and corneal transplants [3]. CXL is the only treatment available to halt progression [5] and is contraindicated in corneas < 400 µm in thickness [1]. Hence it is important to diagnose keratoconic patients early, monitor corneal thickness on a regular basis and refer for CXL when appropriate. CXL strengthens the corneal tissue using ultraviolet-A light and the photosensitizer riboflavin to stiffen the cornea [6] which has been shown to reduce the need for corneal transplantation [7]. When there is documented progression of keratoconus, patients are referred for CXL [3] and where the BCVA does not improve with CLs, patients are referred to an ophthalmologist for other surgical options [8]. The majority of eye care services in Kenya are provided by MLOWs, who are the ophthalmic clinical officers (OCOs) and optometrists. They are trained to conduct refractions, screen for eye diseases, manage and refer when appropriate. Hence, they could play a critical role in the diagnosis and management of keratoconus. The optometrists are restricted to prescribing lubricants and anti-allergy eye drops and do not perform any surgeries, whilst OCOs do not have any restrictions in prescribing eye medication and can perform eye surgeries, including CXL, depending on their respective levels of training and qualifications. Optometrists register with the Kenya Association of Opticians or Optometrists Association of Kenya and OCOs register with the Ophthalmic Clinical Officers Association (OCOA).

In early keratoconus, there may be no obvious changes in corneal structure. Slit lamp signs are mostly seen in moderate-to-severe stages of the disease resulting in the majority of these patients, importantly pediatric patients, being undiagnosed at disease onset [9]. As a progressive disease, a delay in diagnosis has a negative impact on the vision-related quality of life (VR-QOL), education, economic and social aspects; making the early diagnosis a key to halting the progression and preventing visual loss [10].

Little is known about how MLOWs manage keratoconus in Kenya and there are no national guidelines for its diagnosis and management. The study aimed to determine the current clinical practice, referral patterns and barriers faced by practitioners and patients. This will inform national guidelines to promote early diagnosis and management; aimed at preventing progression and ultimately avoiding the need for corneal transplants. Similar studies have been conducted in Australia [11], the UK and Spain [12], Portugal [13] Latin America [14] and South Africa [15]. This is the first study of eye care practices related to keratoconus patient management in Kenya.

## Method

This study used a quantitative, cross-sectional, descriptive design. An online questionnaire, based on previous questionnaires [11, 12] was piloted, modified and distributed via professional associations to 213 OCOs and 282 optometrists over 6 weeks from 13^th^ September to 28^th^ October 2021. The questionnaire comprised 29 questions on practitioner qualifications, experience, location and type of work setting, clinical practice, knowledge, skills and barriers; with an emphasis on the assessment and management of patients with keratoconus (Appendix A in the Supplementary material). The 203 responses were weighted to reflect the total number of OCOs and optometrists in Kenya. This paper presents the results of the questions related to clinical practice.

## Statistical analysis

Descriptive analyses were performed using Stata ‘Svy’ (Stata Corp 17.0) commands to allow for adjustments for the sampling weight when estimating the count and percentages of each category. Cross tabulations were generated to describe the frequencies and confidence intervals of association between dependent and independent variables, and the statistical significances were tested using the chi-squared test and *P* < 0.05 were considered as statistically significant.

## Results

### Demographic information

As shown in Table 1, more registered optometrists (53.5%) than OCOs (24.4%) responded. Twenty-four responses, received from respondents who were not OCOs or optometrists, do not practice in Kenya currently and/or did not disclose the training institution where they qualified, were excluded. A total of 203 responses from 52 OCOs (25.6%) and 151 (74.4%) optometrists were analyzed. The data was weighted to reflect the OCO and optometrist population before being analyzed.

**Table 1:** The percentage of respondents according to the profession

	OCO	Optometrists
No. registered with respective associations (n)	213	282
No. responded to the survey (n)	52	151
% response from each profession	24.4%	53.5%

A significantly higher proportion of OCOs compared to optometrists were trained in Kenya (98.1% OCOs, 82.8% optometrists; *p* = *0.01*), had 10 or more years of experience (38.5% OCOs, 19.9% optometrists, *p* = *0.01*), saw more than 150 patients a month (61.5% OCOs, 24.5% optometrists; *p* < *0.01*), practiced outside the capital city, Nairobi (75.0% OCOs, 44.4% optometrists; *p* < *0.01*) and worked in a hospital setting (98.1% OCOs, 62.3% optometrists; *p* < *0.01*). Less than half of the respondents had a Bachelor’s degree qualification or more (36.5% OCO, 50.3% optometrists; *p* = *0.09*) (Table 2).

**Table 2:** Demographic details of the respondents

Question	OCO (%, 95% CI)	Optometrist (%, 95% CI)	*p*-value
**Which educational institutions have you received qualifications in eye care from?**			
Kenya	98.1 [87.4,99.7]	82.8 [75.8,88.0]	*0.01*
Outside Kenya	1.9 [0.3,12.6]	17.2 [12.0,24.2]	
**What is the highest qualification in eye care that you have achieved?**			
Higher Diploma or less	63.5 [49.6,75.4]	49.7 [41.7,57.7]	*0.09*
Bachelor’s degree or more	36.5 [24.6,50.4]	50.3 [42.3,58.3]	
**How long have you been practicing since you qualified as an eye care practitioner?**			
< 5 years	42.3 [29.6,56.1]	42.5 [34.7,50.5]	*0.01*
5–10 years	19.2 [10.6,32.3]	37.7 [30.3,45.8]	
10 + years	38.5 [26.2,52.3]	19.9 [14.2,27.1]	
**Which city/town do you practice in?**			
Practice in Nairobi	25.0 [15.0,38.6]	55.6 [47.6,63.4]	< *0.01*
Practice outside Nairobi	75.0 [61.4,85.0]	44.4 [36.6,52.4]	
**Which setting do you work in?**			
Optical shop	1.9 [0.3,12.6]	37.7 [30.3,45.8]	< *0.01*
Hospital setting	98.1 [87.4,99.7]	62.3 [54.2,69.7]	

### Diagnosis of keratoconus

**Fig. 1:**
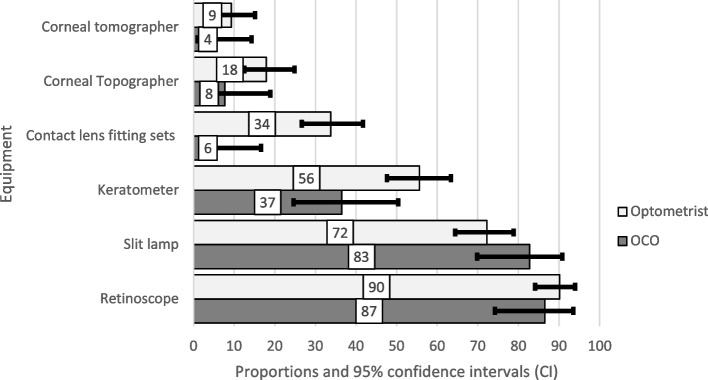
Equipment available to respondents to diagnose and manage keratoconus

The majority of the respondents had access to a retinoscope (88.5%; *p* = *0.48*), performed retinoscopy (86.4%; *p* = *0.06*), had access to (76.7%; *p* = *0.14*) and used slit lamps regularly (79.42%; *p* = *0.18*) but, only a few had access to corneal topographers (13.5%; *p* = *0.08*) or tomographers (6.9%; *p* = *0.21*) (Fig. 1).

**Fig. 2:**
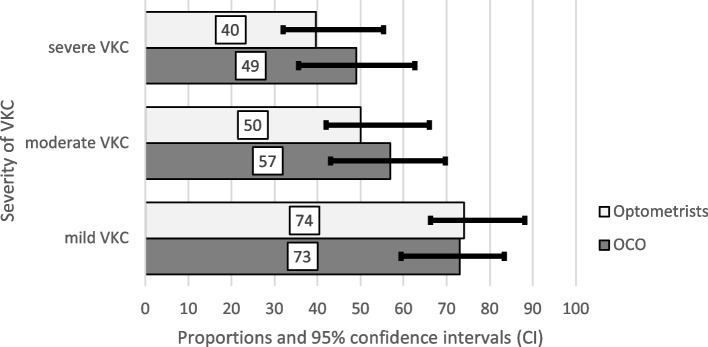
Respondents that advice against eye rubbing in patients with VKC according to disease severity

One-third of the respondents did not consider retinoscopy (38.7%; *p* = *0.21*), slit lamp findings (30.3%; *p* = *0.10*) or corneal topography (36.6%; *p* = *0.39*) important investigations in keratoconus diagnosis. The majority of respondents reported seeing less than or equal to ten keratoconus patients a month (90.0%; *p* = *0.15*), counselled against eye rubbing in mild (73.6%; *p* = *0.90*) vernal keratoconjunctivitis (VKC) which dropped to half in moderate (52.9%; *p* = *0.40*) and severe (43.6%; *p* = *0.24*) cases (Fig. 2). Two-thirds did not recommend corneal topography in patients with VKC (59.0%; *p* = *0.33*).

### Management of keratoconus

There was no statistically significant difference in how OCOs and optometrists managed keratoconus patients. Most respondents (70.1%; *p* = *0.11*) monitored progression in mild cases of keratoconus, 50.9% (*p* = *0.54*) in moderate cases and 25.3% (*p* = *0.31*) in advanced cases. Majority of the respondents (90.2%; *p* = *0.95*) prescribed spectacles in mild cases, which decreased to 29% (*p* = *0.97*) in moderate cases and only 1.9% (*p* = *0.05*) in severe cases.

**Fig. 3:**
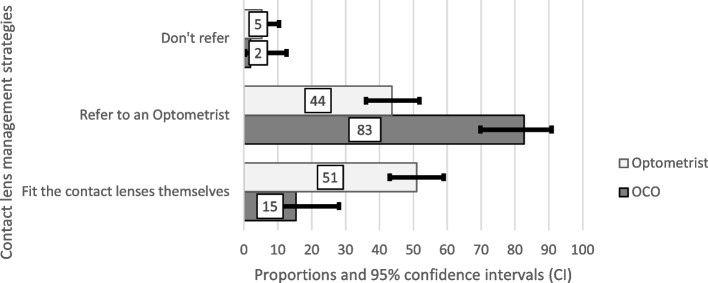
Contact lens management strategies used by respondents

The optometrists had significantly more access to keratometers (36.5% OCOs, 55.6% optometrists; *p* = *0.02*) and CL fitting sets (OCOs 5.8%, optometrists 33.8%; *p* < *0.01*) than the OCO respondents. A small proportion of OCOs fitted rigid CLs (15.4%), compared to optometrists (51.0%; *p* = *0.01*), with the majority referring keratoconus patients to optometrists for CL fitting (82.7% OCOs, 43.7% optometrists; *p* < *0.01*), (Fig. 3). When the binocular BCVA with spectacles was ≤ 6/18, 76.9% of OCOs and 58.9% of optometrists referred keratoconus patients for CLs (*p* = *0.02*). Only 15.4% of OCO respondents performed CXL.

Referrals increased with severity as 30.3% (*p* = *0.57*) of respondents referred keratoconus patients to an ophthalmologist upon initial diagnosis, 50.4% (*p* = *0.35*) referred when there was a reduction in BCVA, 59.8% (*p* = *0.37*) referred on signs of progression and 83.7% (*p* = *0.18*) when the binocular BCVA with CLs dropped to ≤ 6/18. Less than a third of the respondents (26.1%; *p* = *0.10)* referred all keratoconus patients for CXL regardless of age or progression, two-thirds (68.5%; *p* = *0.46*) refer when the condition was progressing and a small percentage (5.4%; *p* = *0.13*) did not know when to refer. Co-management with ophthalmologists after the surgical intervention was reported by 58.0% (*p* = *0.06*) of respondents.

### Associations

Respondents with more than 5 years of experience were more likely to use a slit lamp and a keratometer. There was no clinically significant difference in the use of other instruments, rigid CL fitting and co-management with ophthalmologists based on years of experience. Respondents that worked in the capital city, Nairobi, saw more than ten keratoconus patients a month (*p* = *0.04*), performed keratometry (*p* < *0.01*), pachymetry and corneal tomography (*p* = *0.01*), fitted rigid CLs (*p* = *0.04*) and co-managed keratoconus patients with ophthalmologists (*p* = *0.04*) compared to those working outside the capital city. Those that worked outside Nairobi (*p* = *0.04*) and in hospitals (*p* < *0.01*) were more likely to refer to an optometrist for CL fitting. Those that worked in an optical shop were more likely to fit rigid CLs (*p* < *0.01*). There was no statistically significant difference in the number of keratoconus patients seen per month, the equipment used and the co-management practice based on the work setting i.e. optical shop or hospital (Table 3). A separate analysis between the OCOs and optometrists was conducted, and the results indicated that among the OCOs, we only found an association between co-management and location of practice (see, Supplementary Table 1a for details). Among the Optometrists, we found statistical differences between years of experience and the CL management strategy (*p* = *0.04*), the number of patients seen with keratoconus (*p* = *0.03*) and slit lamp useage (*p* = *0.02*). Among the Optometrists, performing keratometry was strongly linked to years of experience and location of practice with Optometrists in Nairobi reporting a higher prevalence of performing keratometry than Optometrists outside Nairobi (63.1 vs. 41.8) and Optometrists who had 5–10 years’ experience had a higher prevalence than those Optometrists with limited experience (< 5 years). Further details regarding the Optometrists based on work experience, location of practice and type of work setting are reported in Supplementary Table 1b.

**Table 3:** Responses from practitioners based on work experience, location of practice and type of work setting

	Years of experience				Location of practice			Type of work setting		
Question	< 5 years (%, 95% CI)	5–10 years (%, 95% CI)	> 10 years (%, 95% CI)	*p*- value	Nairobi (%, 95% CI)	outside Nairobi (%, 95% CI)	*p*-value	optical shop (%, 95% CI)	hospital (%, 95% CI)	*p*-value
**Patients seen with keratoconus**										
≤ 10/ month	95.4 [87.4,98.4]	84.8 [74.7,91.3]	87.3 [74.3,94.2]	*0.10*	84.7 [76.0,90.7]	93.8 [86.9,97.2]	*0.04*	91.6 [81.1,96.5]	89.5 [83.4,93.6]	*0.66*
> 10/ month	4.6 [1.6,12.6]	15.2 [8.7,25.3]	12.7 [5.8,25.7]	*0.10*	15.3 [9.3,24.0]	6.2 [2.8,13.1]	*0.04*	8.4 [3.5,18.9]	10.5 [6.4,16.6]	*0.66*
**Assessments performed regularly**										
Retinoscopy	87.9 [78.0,93.7]	86.4 [72.3,93.9]	84.3 [70.3,92.4]	*0.87*	89.9 [80.9,94.9]	83.9 [74.3,90.4]	*0.26*	88.2 [77.0,94.3]	85.9 [78.1,91.3]	*0.69*
Slit lamp	69.6 [58.3,78.9]	79.5 [67.9,87.6]	94.3 [82.4,98.3]	< *0.01*	72.8 [62.5,81.1]	84.3 [75.6,90.3]	*0.06*	78.0 [65.6,86.9]	79.8 [72.1,85.8]	*0.78*
Keratometry	32.0 [22.6,43.2]	59.5 [46.4,71.4]	44.9 [31.0,59.7]	*0.01*	56.9 [46.0,67.0]	34.2 [25.3,44.3]	< *0.01*	55.7 [42.5,68.2]	40.4 [32.2,49.1]	*0.05*
Pachymetry	10.0 [5.2,18.2]	16.5 [9.6,26.8]	15.7 [7.6,29.7]	*0.46*	20.8 [13.6,30.5]	8.1 [4.2,15.2]	*0.01*	11.8 [5.7,23.0]	14.0 [9.2,20.7]	*0.68*
Corneal topography	21.0 [13.4,31.3]	25.6 [16.4,37.5]	18.4 [9.6,32.4]	*0.65*	28.1 [19.6,38.4]	16.9 [10.8,25.4]	*0.06*	20.3 [11.8,32.6]	22.0 [15.9,29.7]	*0.78*
Corneal tomography	16.6 [9.8,26.6]	12.7 [6.8,22.3]	5.7 [1.7,17.6]	*0.18*	19.9 [12.8,29.5]	6.8 [3.2,13.8]	*0.01*	10.1 [4.6,20.9]	13.0 [8.4,19.6]	*0.57*
**CL management strategy**										
Fit the CLs yourself	38.3 [28.1,49.5]	38.7 [27.3,51.6]	28.4 [18.2,41.4]	*0.60*	44.1 [34.0,54.6]	29.5 [21.5,38.9]	*0.04*	54.1 [41.0,66.6]	30.4 [23.3,38.6]	< *0.01*
Refer to an optometrist	56.4 [45.1,67.1]	58.7 [45.9,70.4]	68.6 [54.9,79.7]	*0.60*	50.4 [39.9,60.9]	67.9 [58.3,76.2]	*0.04*	40.9 [28.7,54.2]	66.1 [57.8,73.6]	< *0.01*
Don't refer	5.3 [2.4,11.6]	2.5 [0.6,9.7]	3.0 [0.4,18.4]	*0.60*	5.5 [2.2,13.3]	2.6 [1.0,6.9]	*0.04*	5.1 [1.6,14.7]	3.5 [1.5,8.0]	< *0.01*
**Co-management**										
Regularly	29.5 [20.3,40.9]	36.0 [25.0,48.6]	43.0 [29.1,58.1]	*0.30*	45.8 [35.5,56.5]	27.4 [19.3,37.2]	*0.03*	32.4 [21.3,46.0]	36.0 [28.1,44.7]	*0.10*
Occasionally	25.8 [17.2,36.9]	25.8 [17.2,36.9]	13.5 [7.2,24.1]	*0.30*	21.5 [14.4,30.9]	23.8 [16.4,33.3]	*0.03*	33.8 [22.8,46.9]	19.7 [13.7,27.4]	*0.10*
never	44.7 [33.7,56.2]	36.7 [25.2,49.9]	43.5 [29.4,58.7]	*0.30*	32.7 [23.6,43.3]	48.8 [38.6,59.1]	*0.03*	33.8 [22.8,46.9]	44.3 [35.7,53.3]	*0.10*

## Discussion

The responses to the questionnaire were three times higher among optometrists as compared with OCOs. The low response rate from OCOs is possibly due to the survey emanating from a different profession, which may have been thought not to be relevant. Majority of the respondents in both professions had trained in Kenya and hence the study results are only applicable to that context.

Whilst the majority of respondents had access to a retinoscope (88.5%) and a slit lamp (77.5%), very few had access to a corneal topographer (13.5%) and even fewer to a corneal tomographer (6.9%). In its earliest stages, keratoconus can mimic simple refractive errors where there may be no slit lamp signs or changes in corneal thickness and detection is unlikely unless corneal imaging is performed. Corneal topography provides an analysis of the anterior surface only. Corneal tomography provides and analysis of the anterior and posterior corneal surfaces and of corneal thickness which increases sensitivity and specificity for detecting corneal ectasia [1]. Hence, corneal tomography is considered the gold standard for diagnosing keratoconus [3]. The lack of topographers means that many keratoconus patients may not be diagnosed, monitored for progression and/ or referred for CXL promptly. In Spain, Australia, the UK, Latin America and Ghana, 59.8%, 46.4%, 38.1%, 23% and 1.2% of optometrists respectively had access to a corneal topographer [11, 12, 14, 16]. However, corneal topographers and tomographers, are not manufactured locally, are expensive and not easily accessible to practitioners in low-middle-income countries like Kenya, negatively impacting patient care.

A split/scissor retinoscopy reflex was seen in 64% to 100% of keratoconic eyes [4, 17, 18]. Since keratoconus in its early stages mimics a refractive error and retinoscopy is a sensitive and reliable test for detecting keratoconus in early disease [19, 20], MLOWs should be encouraged to check for the presence of a split/scissors reflex in all patients younger than 30 years. A split/ scissor reflex and/or corneal signs for keratoconus should raise suspicion and trigger a referral for corneal imaging. Corneal imaging with a topographer as a minimum, or ideally a tomographer, is required to diagnose and monitor progression in keratoconus [3].

One-third of the respondents did not consider retinoscopy, slit lamp signs and corneal topography important investigations in keratoconus diagnosis, the majority only monitor progression in mild cases and more than half did not recommend corneal topography to their patients with VKC, highlighting a knowledge gap. As keratoconus progresses, VR-QOL reduces significantly. Keratoconus appears in young adulthood and can have lifelong implications, impacting career, education, livelihood and social integration [10]. Hence, it is important to detect keratoconus in its earliest stages, monitor for progression regardless of severity and refer for CXL when there is documented clinical progression [3]. Keratoconus suspects under the age of 30 years should be referred for corneal topography/ tomography [11] and monitored on a regular basis. As keratoconus can progress more rapidly in children, they should be monitored more frequently than adults [3].

Keratoconus is a well-known complication of VKC [21]. Totan et al. [22] and Mugho et al. [23] concluded that the higher incidence of keratoconus in patients with VKC of 26.8% and 30.9% respectively in their studies was due to early detection with corneal topography, emphasizing its importance. Since allergic disorders may be associated with early onset of keratoconus, MLOWs should be encouraged to look for a split/scissor reflex on retinoscopy, slit lamp signs of keratoconus and recommend corneal topography in all patients with VKC [24], especially children [9]. The majority of the respondents only counselled against eye rubbing in mild cases of VKC with approximately half counseling against eye rubbing in moderate and severe cases. Eye rubbing is considered a factor in causing keratoconus progression by the mechanical route and the rise of inflammatory mediators [25]. Patients should be counseled regardless of the severity of VKC on the importance of not rubbing one’s eyes and the use of preservative-free anti-allergy and lubricating eye drops to decrease the impulse of eye rubbing [3].

The majority of the respondents prescribed spectacles only in mild cases of keratoconus. Keratoconic eyes with moderate to severe disease can present with a confusing split/scissor retinoscopy reflex and the auto-refractor may give errors due to the irregular shape of the cornea. With regular practice, practitioners can improve their retinoscopy skills to neutralize the reflex and get a meaningful starting point for subjective refraction. Patients can be allowed to rotate the cylinder axis and bracketing in large dioptric steps can be used. Subjective refraction should be attempted in all patients with ectasia [3]. Only a few OCOs fit rigid CLs and mostly refer keratoconus patients to optometrists for rigid CL fitting. Only half of the optometrists fitted rigid CLs, which are not available locally, making the cost of corneal and scleral lenses prohibitive for the majority of patients. In Kenya, keratoconus is seen in children as young as 6 years old [26]. As younger children may find CLs difficult to manage and uncomfortable, it is even more important to be able to spend additional time doing a subjective refraction to obtain the best possible visual acuity with a spectacle lens. Keratoconus is an asymmetric disease [17] with acuities in one eye being better than in the other eye. In instances where the patient is not able to access, afford, manage or tolerate rigid CLs at the time, the practitioner should aim to achieve the BCVA in at least one eye with spectacles, regardless of the severity of the condition.

The tear film between the rigid CL and the anterior surface of the cornea, neutralizes the irregular keratoconic cornea providing a better BCVA and an improved quality of life compared to spectacles [27, 28]. More optometrist respondents have access to keratometers, CL fitting sets and fit rigid CLs as compared to OCOs. However, the majority of both professions had access to a slit lamp. There is no manufacturing facility for CLs or CL solutions in the country with South Africa being the only known country manufacturing rigid CLs in sub-Saharan Africa [29]. Local manufacturing of rigid CLs and CL solutions across Africa would make them more affordable and accessible.

The majority of the respondents referred keratoconus patients for CL fitting to optometrists when the binocular BCVA was ≤ 6/18 with spectacles and to an ophthalmologist for surgical interventions when the binocular BCVA was ≤ 6/18 with CLs. Rigid CLs should be considered when the vision with spectacles is not satisfactory and surgical interventions when the vision with rigid CLs is not satisfactory [3].

Only a small percentage of OCOs performed CXL. With training and support, nurse-led CXL has been found to be safe in the UK, Ireland and New Zealand [5, 30]. To increase access to CXL, institutions that train OCOs and OCOA should partner with eye hospitals that offer CXL to introduce it into their curricula, and offer post-qualification training.

Referrals took place between the respondents and ophthalmologists for surgical intervention and the majority of OCOs referred their keratoconus patients to optometrists for CL fitting. The rate of co-management between MLOWs and ophthalmologists after surgical interventions in keratoconus patients in Kenya (58.0%) is similar to that in Australia (58.8%) and much higher than in Latin America (40.4%), the UK (39.7%), Spain (27.2%) and Portugal (17.1%) [11–14]. After surgical interventions such as CXL, ICRS or corneal transplants, vision rehabilitation with spectacles or CLs is still required [3] and hence it is important for ophthalmologists to co-manage keratoconus patients with MLOWs to improve visual outcomes. The need for improving interdisciplinary collaboration and the development of clinical guidelines in keratoconus diagnosis and management has been highlighted across the globe in four similar studies [11–14].

Location may be a factor with MLOWs practicing in Nairobi seeing more patients with keratoconus, the majority of whom are likely to have travelled from another town. To strengthen the eye health system towards universal health coverage, epidemiological studies that provide the prevalence of the disease, based on geographical location and other demographic strata, are essential.

Practitioners who worked in the capital city had access to more equipment such as keratometers, pachymeters and tomographers, were able to offer rigid CL fitting services and had more co-management opportunities than their colleagues who worked outside Nairobi. This is because there are more secondary and tertiary eye care services in the capital city, mostly dominated by the private sector. Eye care services outside Nairobi are mostly provided by public hospitals which do not offer CL fitting services and hence most practitioners who work outside Nairobi, and in hospitals, refer keratoconus patients to an optometrist for rigid CL fitting. There was no significant difference in the number of practitioners fitting or referring for rigid CLs between the younger and more experienced MLOWs, suggesting a similar understanding of the disease. This reflects a possible lack of continuous education opportunities in advanced fitting techniques, which one would have expected the more experienced practitioners to have and practice. Further training could result in more optometrists fitting rigid CLs [12]. Mapping of existing services for keratoconus patients in both the public and private sector and professional development opportunities would highlight the service and knowledge gaps to inform recommendations made to the relevant health sector stakeholders.

A limitation of this study was the low number of responses. However, a strength of this study is that it is the first to analyze and make recommendations on the current practice in keratoconus diagnosis and management amongst MLOWs in Kenya.

## Conclusion

If keratoconus is identified early, progression monitored and patients referred for CXL when necessary, the number of patients who require rigid CLs or surgical interventions can drastically reduce. Our findings reflect a lack of diagnostic tools and a gap in knowledge in the diagnosis and management of keratoconus amongst MLOWs in Kenya. There is a need for cost–benefit analysis studies to empirically determine the economic implications of keratoconus on the health system to make suitable recommendations to increase affordability and access to care. The gaps in knowledge and clinical practice can be used by training institutions and professional associations to review current curricula and set national standards for both undergraduate education and continuous professional education. Additionally, relevant stakeholders could collaborate to develop keratoconus diagnosis and management guidelines for clinical practice in both private and public sectors. There is a need for greater interdisciplinary co-management between ophthalmologists and MLOWs to improve visual outcomes in patients with keratoconus.

## Supplementary Information

**Additional file 1.**

**Additional file 2:Supplementary Table 1a.** Responses from OCOs based on work experience, location of practice and type of work setting, **Supplementary Table 1b.** Responses from optometrists based on work experience, location of practice and type of work setting.

## Data Availability

All data generated or analyzed during this study are included in this published article [and its supplementary information files].

## Abbreviations

BCVA: Best corrected visual acuity
CL: Contact lens
CXL: Corneal cross-linking
MLOW: Mid-level ophthalmic worker
OCO: Ophthalmic Clinical Officer
OCOA: Ophthalmic Clinical Officer’s Association
VKC: Vernal keratoconjunctivitis
VR-QOL: Vision-related quality of life
