# Supplementary material for: Diagnosis and management of keratoconus by eye care practitioners in Kenya
Source: BMC Ophthalmol. 2023 Jan 27;23:37. doi: 10.1186/s12886-023-02792-w (PMC9881246; doi:10.1186/s12886-023-02792-w)
Supplement: Supplementary file 1 — Additional file 1. [file 12886_2023_2792_MOESM1_ESM.pdf]

Keratoconus Management: a survey of primary eye care practice in Kenya

Dear Eye Care Practitioner

This survey is intended for Ophthalmic Clinical Officers, Opticians and Optometrists practicing in Kenya.

In order to to develop national guidelines on the screening, diagnosis and management of keratoconus, we need to establish how keratoconus patients are managed in the primary eye care setting in Kenya. The survey should not take more than 15 minutes and is accessible on your phone.

Your return of this anonymous survey implies your consent to participate in this research. Your contribution to this research is appreciated.

For further information please contact Zahra Rashid (email: alyzahra@gmail.com, Tel: 0734726600).

The study has received ethical approval from Ministry of Health - Office of the Director General Ref: MOH/ADM/1/1/82(100), Amref Health Africa, Ref: ESRC P918/2021 and the Biomedical Research Ethics Administration (BREC), University of Kwazulu-Natal (UKZN), Durban South Africa Ref: BREC/00001226/2020. Should you have any concerns about the conduct of this research project, you can contact:
a) Ministry of Health - Office of the Director General, Mobile no. 020-2717077, email: dghealth2019@gmail.com
b) The Research Officer, Amref Health Africa in Kenya, Office Tel: 020-6994000, Mobile No: 0795746777,OR
c) Dr. J Moodley, BREC, UKZN, Tel: (031) 260 4604, Email: jmog@ukzn.ac.za

Thank you for your support.

Zahra Rashid
PhD student
Department of Optometry
University of Kwazulu-Natal,
Durban, South Africa

*Required

1. 1. Choose your qualification *

Mark only one oval.

- ( ) Ophthalmic Clinical Officer
- ( ) Optician
- ( ) Optometrist
- ( ) Other: ____________

2. 2. Which educational institutions have you received qualifications in eye care from? *

Tick all that apply.

- [ ] KMTC
- [ ] Masinde Muliro University of Science and Technology
- [ ] Other: ____________________

3. 3. What is the highest qualification in eye care that you have achieved? *

Mark only one oval.

- ( ) Diploma
- ( ) Higher Diploma
- ( ) Bachelor
- ( ) Masters
- ( ) Doctorate
- ( ) PhD

4. 4. How long have you been practicing since you qualified as an Eye Care Practitioner? *

Mark only one oval.

- ( ) <5 years
- ( ) 5-10 years
- ( ) 10-15 years
- ( ) 15-20 years
- ( ) >20 years

5. 5. Which City/Town do you practice in? *

____

6. 6. Which setting do you work in? *

Tick all that apply.

- [ ] Optical Shop
- [ ] Public Hospital/Clinic
- [ ] Private Hospital/Clinic
- [ ] NGO/Faith based Hospital
- [ ] University/ College
- [ ] Other: ________________

7. 7. Please select the following equipment/ consumables that you have access to at your place of work: *

Tick all that apply.

- [ ] Distance visual acuity charts
- [ ] Retinoscope
- [ ] Trial lens set and trial frame
- [ ] Lens meter/ Focimeter
- [ ] Cross-cylinder
- [ ] Auto-refractor
- [ ] Slit lamp
- [ ] Keratometer
- [ ] Corneal topographer
- [ ] Pachymeter
- [ ] Corneal tomographer
- [ ] Local anesthetic drops
- [ ] Fluorescein
- [ ] Contact lens fitting sets for keratoconic eyes
- [ ] Contact lens solutions

8. 8. Do you perform the following assessments regularly? *

Mark only one oval per row.

	Yes	No
Retinoscopy		
Subjective refraction		
Slit lamp exam		
Keratometry		
Pachymetry		
Corneal topography		
Corneal tomography		
Fit hard corneal contact lenses		
Fit hybrid/scleral contact lenses		
Manage mild allergic conjunctivitis		
Manage moderate-severe allergic conjunctivitis		
Corneal cross-linking		

9. 9. Are you aware of national guidelines on the diagnosis and management of the following eye conditions? *

Mark only one oval per row.

	Yes	No
Allergic conjunctivitis		
Keratoconus		

10. 10. How many patients do you see on a monthly basis? *

Mark only one oval.

- <50
- 50-100
- 100-150
- 150-200
- >200

11. 11. When you see a patient with allergic conjunctivitis do you perform/recommend the following investigations? *

Mark only one oval per row.

	Always	sometimes	never
**Refraction**			
**Slit lamp exam**			
**Keratometry**			
**Corneal topography**			

12. 12. How do you therapeutically manage patients with allergic conjunctivitis?

Tick all that apply.

	mild cases	moderate cases	severe cases
**Counsel against eye rubbing**			
**Prescribe a lubricant**			
**Prescribe an anti-allergy eye drop**			
**Prescribe a steroid eye drop**			
**Refer to an ophthalmologist**			

13. 13. How many patients on average with keratoconus do you see in a month? *

Mark only one oval.

- ( ) None
- ( ) 1-10
- ( ) 11-20
- ( ) >20

14. 14. What investigations would you consider important in making a diagnosis of keratoconus? *

Tick all that apply.

- [ ] History and visual acuity
- [ ] Retinoscopy
- [ ] Manual Keratometry
- [ ] Slit lamps signs
- [ ] Corneal topography/tomography
- [ ] Other: ______________________

15. 15. How do you manage your keratoconus (KC) patients? *

Tick all that apply.

	mild KC	moderate KC	advanced KC
**Prescribe spectacles**	[ ]	[ ]	[ ]
**Fit/refer corneal hard contact lenses**	[ ]	[ ]	[ ]
**Fit/refer hybrid/scleral contact lenses**	[ ]	[ ]	[ ]
**manage/refer allergic conjunctivitis**	[ ]	[ ]	[ ]
**monitor progression**	[ ]	[ ]	[ ]
**Refer to ophtalmologist**	[ ]	[ ]	[ ]

16. 16. What is the lower limit of binocular best corrected visual acuity in glasses that you would fit/refer a patient with keratoconus for specialized contact lenses? *

Mark only one oval.

- 6/6
- 6/9
- 6/12
- 6/18
- 6/24
- 6/60

17. 17. What is the lower limit of binocular best corrected visual acuity with contact lenses that you would refer a patient with keratoconus to an ophthalmologist for possible surgical interventions? *

Mark only one oval.

- 6/6
- 6/9
- 6/12
- 6/18
- 6/24
- 6/60

18. 18. How do you manage Keratoconus patients who require contact lenses?

Mark only one oval.

- Fit the contact lenses yourself
- Refer to an optometrist
- Don't refer

19. 19. At what stage would you consider referring a patient with keratoconus to an ophthalmologist? *

Tick all that apply.

- [ ] Upon initial diagnosis
- [ ] At patients request
- [ ] Signs and symptoms of mild allergic conjunctivitis
- [ ] Signs and symptoms of moderate to severe allergic conjunctivitis
- [ ] Reduction of best corrected visual acuity
- [ ] Signs of progression
- [ ] No set time

20. 20. Which patients do you recommend for corneal cross-linking? *

Mark only one oval.

- ( ) All keratoconus patients regardless of age and whether the condition is progressing or stable
- ( ) Only those keratoconus patients whose condition is progressing
- ( ) I don't know
- ( ) Other: ____________________

21. 21. Do you currently co-manage patients with ophthalmologists after surgical treatment, for example: corneal cross-linking or contact lens fitting following intra-stromal corneal rings or penetrating keratoplasty? *

Mark only one oval.

- ( ) Yes
- ( ) No
- ( ) occasionally

22. 22. At the Practice/Hospital/Clinic where you work: *

Mark only one oval per row.

	Yes	No
**Do you have colleagues in eye care with whom you can share knowledge and experiences**	◯	◯
**Do you have access to continuous medical education in eye care**	◯	◯

23. 23. Which of the following options do you use to update your knowledge and skills in eye care? *

Tick all that apply.

- [ ] Journals/publications/online resources
- [ ] Attending continuous medical education sessions
- [ ] Registering for courses
- [ ] Attending conferences
- [ ] Other: ____________________

24. 24. On a scale of 1-5 on how confident do you feel at using the following tools/performing the following assessments when screening, diagnosing and managing patients with keratoconus (1-not confident, 5- very confident) *

Mark only one oval per row.

	1	2	3	4	5
**Retinoscopy**	⬭	⬭	⬭	⬭	⬭
**Subjective Refraction**	⬭	⬭	⬭	⬭	⬭
**Keratometry**	⬭	⬭	⬭	⬭	⬭
**Corneal Topography**	⬭	⬭	⬭	⬭	⬭
**Hard contact lens fitting**	⬭	⬭	⬭	⬭	⬭
**Managing mild allergic conjunctivitis**	⬭	⬭	⬭	⬭	⬭
**Managing moderate allergic conjunctivitis**	⬭	⬭	⬭	⬭	⬭

25. 25. Please rate your interest for further training in the following areas (1- not interested, 5 very interested) *

Mark only one oval per row.

	1	2	3	4	5
**Keratoconus diagnosis & management**	⬭	⬭	⬭	⬭	⬭
**Retinoscopy**	⬭	⬭	⬭	⬭	⬭
**Subjective Refraction**	⬭	⬭	⬭	⬭	⬭
**Corneal topography**	⬭	⬭	⬭	⬭	⬭
**Hard contact lens fitting**	⬭	⬭	⬭	⬭	⬭
**Managing allergic conjunctivitis**	⬭	⬭	⬭	⬭	⬭
**Corneal cross-linking**	⬭	⬭	⬭	⬭	⬭

26. 26. Do the following factors hinder you from increasing your knowledge and skills in eye care? *

Mark only one oval per row.

	Always	Sometimes	Never
**Not knowing where to access it**	⬭	⬭	⬭
**No mentor at work**	⬭	⬭	⬭
**Lack of diagnostic tools**	⬭	⬭	⬭
**Lack of professional development opportunities**	⬭	⬭	⬭
**Cost of conferences**	⬭	⬭	⬭
**Cost of journal subscriptions**	⬭	⬭	⬭
**Lack of motivation / incentive**	⬭	⬭	⬭
**Time constraints**	⬭	⬭	⬭

27. 27. Do the following factors hinder you from diagnosing keratoconus (KC) early and managing your patients effectively? *

Mark only one oval per row.

	Yes	No
**Lack of knowledge & skills**	◯	◯
**Lack of useable equipment**	◯	◯
**Cost of diagnostic equipment**	◯	◯
**Difficulty in examining eyes with KC**	◯	◯
**Lack of facilities that offer KC services in the same city where you work**	◯	◯
**Lack of spectacle supply**	◯	◯
**Cost of spectacles**	◯	◯
**Lack of local hard contact lens supply**	◯	◯
**Cost of hard contact lenses**	◯	◯
**Lack of national guidelines on managing KC**	◯	◯
**Lack of patient educational material**	◯	◯
**Lack of space**	◯	◯
**Fear of litigation**	◯	◯
**Lack of support from clinic leadership**	◯	◯
**Poor communication with secondary eye care facilities**	◯	◯

28. 28. Do the following patient factors act as barriers in screening, diagnosing and managing your keratoconus patients effectively ? *

Mark only one oval per row.

	Always	Sometimes	Never
**Patient affordability**	⬭	⬭	⬭
**Patient motivation**	⬭	⬭	⬭
**Willingness to pay**	⬭	⬭	⬭
**Lack of patient education about KC**	⬭	⬭	⬭
**Language barrier**	⬭	⬭	⬭
**Hard contact lenses can be uncomfortable**	⬭	⬭	⬭
**Patient compliance**	⬭	⬭	⬭
**Patient perception of your role**	⬭	⬭	⬭

29. 29. Please list any barriers not covered in the questions above that hinder you from providing quality eye care services to your keratoconus patients

This content is neither created nor endorsed by Google.

Google Forms
