# Supplementary material for: Diagnosis and management of keratoconus by eye care practitioners in Kenya
Source: BMC Ophthalmol. 2023 Jan 27;23:37. doi: 10.1186/s12886-023-02792-w (PMC9881246; doi:10.1186/s12886-023-02792-w)
Supplement: Supplementary file 2 — Additional file 2:Supplementary Table 1a. Responses from OCOs based on work experience, location of practice and type of work setting, Supplementary Table 1b. Responses from optometrists based on work experience, location of practice and type of work setting. [file 12886_2023_2792_MOESM2_ESM.docx]

| **Supplementary Table 1a** Responses from OCOs based on work experience, location of practice and type of work setting | | | | | | | | |  |  |
| --- | --- | --- | --- | --- | --- | --- | --- | --- | --- | --- |
|  | Years of experience | | | | Location of practice | | | Type of work setting | | |
| Question | <5 years (%, 95% CI) | 5-10 years (%, 95% CI) | >10 years (%, 95% CI) | *p*- value | Nairobi (%, 95% CI) | outside Nairobi (%, 95% CI) | *p*-value | optical shop (%, 95% CI) | hospital (%, 95% CI) | *p*-value |
| **Patients seen with keratoconus** |  |  |  |  |  |  |  |  |  |  |
| ≤10/ month | 95.5 [72.5,99.4] | 100.0 | 90.0 [66.5,97.6] | *0.52* | 92.3 [59.3,99.0] | 94.9 [80.9,98.8] | *0.74* | 100 | 94.1 [82.7,98.2] | *0.80* |
| >10/ month | 4.5 [0.6,27.5] | 0.0 | 10.0 [2.4,33.5] |  | 7.7 [1.0,40.7] | 5.1 [1.2,19.1] |  | 0 | 5.9 [1.8,17.3] |  |
| **Assessments performed regularly** |  |  |  |  |  |  |  |  |  |  |
| Retinoscopy | 86.4 [64.3,95.7] | 60.0 [28.8,84.7] | 85.0 [61.4,95.3] | *0.19* | 84.6 [53.7,96.3] | 79.5 [63.4,89.6] | *0.69* | 100 | 80.4 [66.7,89.3] | *0.62* |
| Slit lamp | 72.7 [50.3,87.6] | 90.0 [51.5,98.7] | 95.0 [70.4,99.3] | *0.13* | 76.9 [46.7,92.7] | 87.2 [72.0,94.7] | *0.38* | 100 | 84.3 [71.1,92.1] | *0.67* |
| Keratometry | 22.7 [9.5,45.2] | 50.0 [21.7,78.3] | 30.0 [13.7,53.5] | *0.31* | 38.5 [16.4,66.5] | 28.2 [16.0,44.7] | *0.50* | 0 | 31.4 [19.9,45.7] | *0.50* |
| Pachymetry | 4.5 [0.6,27.5] | 0 | 15.0 [4.7,38.6] | *0.27* | 15.4 [3.7,46.3] | 5.1 [1.2,19.1] | *0.24* | 0 | 7.8 [2.9,19.7] | *0.77* |
| Corneal topography | 13.6 [4.3,35.7] | 10.0 [1.3,48.5] | 15.0 [4.7,38.6] | *0.93* | 23.1 [7.3,53.3] | 10.3 [3.8,25.0] | *0.25* | 0.0 | 13.7 [6.5,26.6] | *0.69* |
| Corneal tomography | 13.6 [4.3,35.7] | 0 | 5.0 [0.7,29.6] | *0.35* | 15.4 [3.7,46.3] | 5.1 [1.2,19.1] | *0.24* | 0 | 7.8 [2.9,19.7] | *0.77* |
| **CL management strategy** |  |  |  |  |  |  |  |  |  |  |
| Fit the CLs yourself | 22.7 [9.5,45.2] | 30.0 [9.6,63.5] | 0.0 | *0.11* | 23.1 [7.3,53.3] | 12.8 [5.3,28.0] | *0.14* | 0 | 15.7 [7.9,28.9] | *0.90* |
| Refer to an optometrist | 77.3 [54.8,90.5] | 70.0 [36.5,90.4] | 95.0 [70.4,99.3] |  | 69.2 [40.0,88.4] | 87.2 [72.0,94.7] |  | 100.0 | 82.4 [68.9,90.8] |  |
| Don't refer | 0.0 | 0.0 | 5.0 [0.7,29.6] |  | 7.7 [1.0,40.7] | 0 |  | 0.0 | 2.0 [0.3,13.4] |  |
| **Co-management** |  |  |  |  |  |  |  |  |  |  |
| Regularly | 27.3 [12.4,49.7] | 20.0 [4.8,55.4] | 45.0 [24.8,67.1] | *0.14* | 61.5 [33.5,83.6] | 23.1 [12.2,39.3] | *0.04* | 100.0 | 31.4 [19.9,45.7] | *0.36* |
| Occasionally | 22.7 [9.5,45.2] | 30.0 [9.6,63.5] | 0 |  | 7.7 [1.0,40.7] | 17.9 [8.6,33.8] |  | 0.0 | 15.7 [7.9,28.9] |  |
| never | 50.0 [29.6,70.4] | 50.0 [21.7,78.3] | 55.0 [32.9,75.2] |  | 30.8 [11.6,60.0] | 59.0 [42.6,73.6] |  | 0.0 | 52.9 [38.9,66.5] |  |

| **Supplementary Table 1b** Responses from optometrists based on work experience, location of practice and type of work setting | | | | | | | | | |  |
| --- | --- | --- | --- | --- | --- | --- | --- | --- | --- | --- |
|  | Years of experience | | | | Location of practice | | | Type of work setting | | |
| Question | <5 years (%, 95% CI) | 5-10 years (%, 95% CI) | >10 years (%, 95% CI) | *p*- value | Nairobi (%, 95% CI) | outside Nairobi (%, 95% CI) | *p*-value | optical shop (%, 95% CI) | hospital (%, 95% CI) | *p*-value |
| **Patients seen with keratoconus** |  |  |  |  |  |  |  |  |  |  |
| ≤10/ month | 95.3 [86.3,98.5] | 78.9 [66.3,87.7] | 83.3 [65.4,93.0] | *0.03* | 82.1 [72.3,89.0] | 92.5 [83.1,96.9] | *0.06* | 91.2 [80.4,96.3] | 84.0 [75.1,90.2] | *0.21* |
| >10/ month | 4.7 [1.5,13.7] | 21.1 [12.3,33.7] | 16.7 [7.0,34.6] |  | 17.9 [11.0,27.7] | 7.5 [3.1,16.9] | *0.06* | 8.8 [3.7,19.6] | 16.0 [9.8,24.9] |  |
| **Assessments performed regularly** |  |  |  |  |  |  |  |  |  |  |
| Retinoscopy | 89.1 [78.6,94.7] | 96.5 [86.8,99.1] | 83.3 [65.4,93.0] | *0.11* | 91.7 [83.4,96.0] | 89.6 [79.5,95.0] | *0.66* | 87.7 [76.2,94.1] | 92.6 [85.1,96.4] | *0.32* |
| Slit lamp | 67.2 [54.7,77.6] | 75.4 [62.5,85.0] | 93.3 [76.6,98.4] | *0.02* | 71.4 [60.8,80.1] | 80.6 [69.2,88.5] | *0.20* | 77.2 [64.4,86.4] | 74.5 [64.6,82.3] | *0.71* |
| Keratometry | 39.1 [27.8,51.6] | 63.2 [49.9,74.7] | 66.7 [48.1,81.2] | *0.01* | 63.1 [52.2,72.8] | 41.8 [30.5,54.0] | *0.01* | 57.9 [44.7,70.1] | 51.1 [40.9,61.1] | *0.42* |
| Pachymetry | 14.1 [7.4,25.0] | 22.8 [13.6,35.6] | 16.7 [7.0,34.6] | *0.45* | 22.6 [14.8,32.9] | 11.9 [6.0,22.2] | *0.09* | 12.3 [5.9,23.8] | 21.3 [14.1,30.8] | *0.17* |
| Corneal topography | 26.6 [17.1,38.8] | 31.6 [20.8,44.8] | 23.3 [11.5,41.7] | *0.69* | 29.8 [20.9,40.5] | 25.4 [16.3,37.2] | *0.55* | 21.1 [12.3,33.7] | 31.9 [23.2,42.1] | *0.15* |
| Corneal tomography | 18.8 [10.9,30.3] | 17.5 [9.6,29.8] | 6.7 [1.6,23.4] | *0.30* | 21.4 [13.9,31.6] | 9.0 [4.0,18.7] | *0.04* | 10.5 [4.8,21.7] | 19.1 [12.3,28.5] | *0.16* |
| **CL management strategy** |  |  |  |  |  |  |  |  |  |  |
| Fit the CLs yourself | 50.0 [37.9,62.1] | 42.1 [29.9,55.3] | 70.0 [51.4,83.7] | *0.04* | 51.2 [40.5,61.8] | 50.7 [38.8,62.6] | *0.95* | 56.1 [43.0,68.5] | 47.9 [37.9,58.0] | *0.60* |
| Refer to an optometrist | 40.6 [29.2,53.1] | 54.4 [41.3,66.9] | 30.0 [16.3,48.6] |  | 44.0 [33.7,54.9] | 43.3 [31.9,55.4] |  | 38.6 [26.8,51.9] | 46.8 [36.9,57.0] |  |
| Don't refer | 9.4 [4.2,19.5] | 3.5 [0.9,13.2] | 0.0 |  | 4.8 [1.8,12.1] | 6.0 [2.2,15.0] |  | 5.3 [1.7,15.3] | 5.3 [2.2,12.3] |  |
| **Co-management** |  |  |  |  |  |  |  |  |  |  |
| Regularly | 31.3 [21.0,43.7] | 42.1 [29.9,55.3] | 40.0 [24.2,58.3] | *0.60* | 40.5 [30.4,51.4] | 32.8 [22.6,45.0] | *0.61* | 100.0 | 31.4 [19.9,45.7] | *0.36* |
| Occasionally | 28.1 [18.4,40.4] | 26.3 [16.4,39.3] | 33.3 [18.8,51.9] |  | 26.2 [17.8,36.7] | 31.3 [21.3,43.5] |  | 0.0 | 15.7 [7.9,28.9] |  |
| never | 40.6 [29.2,53.1] | 31.6 [20.8,44.8] | 26.7 [13.8,45.2] |  | 33.3 [24.0,44.2] | 35.8 [25.2,48.1] |  | 0.0 | 52.9 [38.9,66.5] |  |
